# Towards a Cure for Diamond–Blackfan Anemia: Views on Gene Therapy

**DOI:** 10.3390/cells13110920

**Published:** 2024-05-27

**Authors:** Matilde Vale, Jan Prochazka, Radislav Sedlacek

**Affiliations:** 1 Laboratory of Transgenic Models of Diseases, Institute of Molecular Genetics of the Czech Academy of Sciences, v.v.i, 252 50 Vestec, Czech Republic; ana-matilde.vale@img.cas.cz (M.V.); jan.prochazka@img.cas.cz (J.P.); 2 Czech Centre for Phenogenomics, Institute of Molecular Genetics of the Czech Academy of Sciences, v.v.i, 252 50 Vestec, Czech Republic

**Keywords:** Diamond–Blackfan anemia, ribosomopathy, ribosomal protein genes, rare genetic disorder, hematopoietic stem cell transplantation, gene therapy, lentiviral vector, non-integrating lentiviral vector, CRISPR/Cas9

## Abstract

Diamond–Blackfan anemia (DBA) is a rare genetic disorder affecting the bone marrow’s ability to produce red blood cells, leading to severe anemia and various physical abnormalities. Approximately 75% of DBA cases involve heterozygous mutations in ribosomal protein (RP) genes, classifying it as a ribosomopathy, with RPS19 being the most frequently mutated gene. Non-RP mutations, such as in GATA1, have also been identified. Current treatments include glucocorticosteroids, blood transfusions, and hematopoietic stem cell transplantation (HSCT), with HSCT being the only curative option, albeit with challenges like donor availability and immunological complications. Gene therapy, particularly using lentiviral vectors and CRISPR/Cas9 technology, emerges as a promising alternative. This review explores the potential of gene therapy, focusing on lentiviral vectors and CRISPR/Cas9 technology in combination with non-integrating lentiviral vectors, as a curative solution for DBA. It highlights the transformative advancements in the treatment landscape of DBA, offering hope for individuals affected by this condition.

## 1. Introduction

Diamond–Blackfan anemia (DBA) is a rare inherited bone marrow failure syndrome (IBMFS) characterized by erythroid hypoplasia, primarily affecting infants [1]. This condition, estimated to occur in 5–7 cases per million live births [1], is considered one of the emerging group of disorders known as ribosomopathies [2], which arise from defects in ribosome biogenesis and function. Approximately 75% of DBA cases involve heterozygous mutations in ribosomal protein (RP) genes [1]. In fact, the initial discovery of genetic mutations in DBA was attributed to mutations in the RPS19 gene, which encodes one of the proteins in the 40S small ribosomal subunit [3]. Among the 81 RP-encoding genes, mutations have been identified in 19 of them, with RPS19 (25%), RPL5 (7%), RPS26 (6.6%), and RPL11 (5%) being the most frequently mutated in DBA [2]. Recent advancements have identified mutations in GATA1, a key erythroid transcription factor, as the first non-RP mutations in DBA patients. This discovery followed the identification of other non-RP gene mutations in the RPS26 chaperone protein TSR2 [3].

The current therapeutic strategies for DBA include glucocorticosteroids (GC), blood transfusions, and hematopoietic stem cell transplantation (HSCT), each with its own set of limitations. Glucocorticosteroids, despite being commonly used, may lose effectiveness over time, particularly in patients who become non-responsive to long-term treatment. Moreover, long-term or high-dose therapies with GCs can lead to a range of adverse effects, including osteoporosis, skin atrophy, diabetes, abdominal obesity, glaucoma, cataracts, avascular necrosis and infection, growth retardation, and hypertension [4]. Blood transfusions serve as a vital supportive measure to alleviate symptoms and manage anemia in DBA patients, but it is important to notice that there is a toxicity associated with iron overload [5]. HSCT is the only curative treatment for DBA and although it can be an option for patients with steroid resistance and transfusion dependency, it presents challenges as finding suitable donors and the risk of immunological complications [6]. Amidst these challenges, gene therapy emerges as a promising tool for treating DBA.

Gene therapy holds significant importance in the treatment of DBA due to the limitations of current therapies. Recent advances in gene therapy, particularly the use of lentiviral vectors, show promise for treating DBA. These therapies aim to correct the genetic defects causing DBA by introducing functional copies of the mutated genes into the patient’s cells. As research in this field progresses, there is growing potential for gene therapy to correct the underlying genetic mutations associated with DBA, using techniques such as the CRISPR/Cas9 editing tool [1,3].

In this review, we aim to explore the potential of gene therapy based on CRISPR/Cas9 technology, particularly in combination with non-integrating lentiviral vectors, as a curative solution for DBA. We will delve into how these innovative approaches hold the key to restoring normal hematopoiesis, thereby offering transformative advancements in the treatment landscape of DBA. Continued research and refinement of gene therapy strategies can unlock this potential and dive into a new era of hope for individuals affected by DBA.

## 2. Clinical Presentation of DBA and Diagnosis

**Figure 1:**
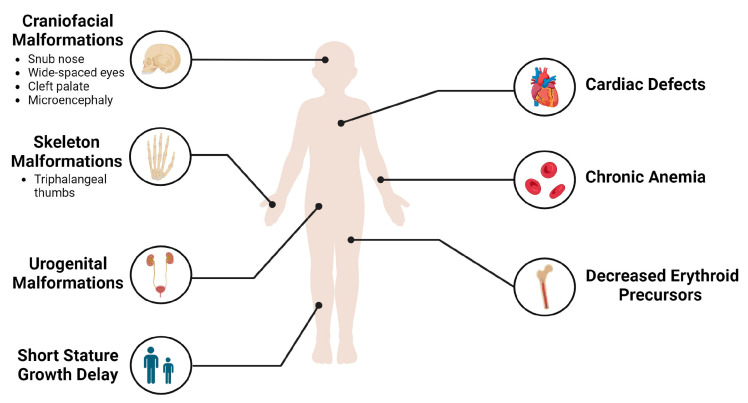
Clinical manifestations of DBA. Created with BioRender.com (accessed on 18 April 2024).

In individuals with DBA, the hematological profile typically shows macrocytic or occasionally normocytic anemia along with reticulocytopenia. Patients usually present normal neutrophil and platelet counts, and the bone marrow appears normal in terms of cellularity but has a deficiency in erythroid precursors [7]. Symptoms of DBA often surface in infancy, with 95% of cases being diagnosed before 2 years of age and 99% before 5 years of age. These symptoms include anemia-related signs such as pallor, fatigue, and feeding difficulties [8]. Although DBA is primarily a hematological disorder, patients also exhibit a spectrum of physical abnormalities. Common features among DBA patients include delayed growth, short stature, and a distinct facial appearance known as Cathie facies, which is characterized by a cute snub nose and wide-spaced eyes. Triphalangeal thumbs, a condition known as Aase syndrome, are also common, and are often accompanied by craniofacial malformations, cleft palate, cardiac defects, and urogenital malformations (Figure 1) [7,9]. DBA patients also face an elevated risk of developing various cancers, including hematological malignancies and solid tumors, such as colon carcinoma and osteosarcomas [1].

In addition to the classic hematological profile of DBA patients, a significant number of non-classic cases have been identified, requiring alternative diagnostic approaches beyond traditional methods, such as complete blood count, reticulocyte count, and bone marrow aspiration and biopsy. Diagnosis typically involves assessing fetal hemoglobin (HbF) levels and erythrocyte adenosine deaminase (eADA) activity, as these are considered biomarkers of DBA [7,10]. When clinical suspicion arises, mutation analysis for known DBA genes is conducted to confirm the diagnosis [7]. These diagnostic strategies allow for comprehensive evaluation and accurate identification of DBA, facilitating appropriate management and care for affected individuals.

## 3. Molecular Mechanism of DBA

The intricate molecular pathways underlying DBA remain incompletely elucidated, motivating ongoing scientific efforts to unravel the relationship between mutations, mostly in RP genes, and the resultant anomalies in ribosome assembly and biogenesis, ultimately culminating in impaired erythropoiesis.

**Figure 2:**
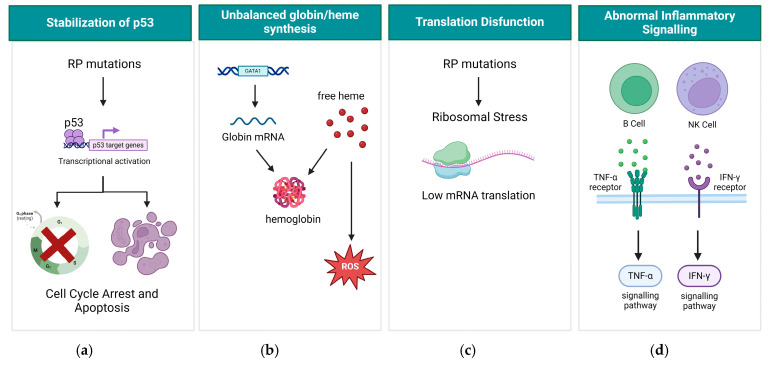
Molecular mechanism of DBA. (**a**) RP mutations lead to activation of p53, cell cycle arrest, and apoptosis; (**b**) Unbalanced globin/heme synthesis leads to accumulation of ROS in erythroid precursors; (**c**) Translation dysfunction caused by RP mutations; (**d**) Abnormal inflammatory signaling caused by RP mutations. Created with BioRender.com (accessed on 22 May 2024).

Research has shed light on one aspect of this complexity, revealing that haploinsufficiency in certain RP genes leads to the stabilization of p53, leading to cell cycle arrest and apoptosis (Figure 2a) [1]. Remarkably, studies using zebrafish and patient samples have shown that mutations in RP genes are related to the activation of p53 and target genes [11,12,13]. Additionally, unbalanced globin/heme synthesis emerges as another critical aspect of DBA pathogenesis. Reports indicate that primary DBA cells exhibit imbalanced globin and heme synthesis, resulting in the accumulation of reactive oxygen species (ROS) within early erythroid precursors. This accumulation significantly contributes to the impairment of erythropoiesis (Figure 2b) [14]. Translational dysfunction also emerges as a key player in DBA pathology (Figure 2c). This occurs when ribosomal stress, induced by mutations in RP genes, leads to issues in protein synthesis. It is possible that ribosome dysfunction can affect mRNA production and that certain specific cells or tissues may be more vulnerable to ribosome dysfunction [15]. Notably, in patients with RP mutations, the mRNA for GATA1, a master hematopoietic transcription factor, is poorly translated, further exacerbating the impaired erythroid defect characteristic of DBA, which might be due to the fact that this mRNA has a higher threshold for initiation in comparison to other mRNAs [16]. Moreover, emerging evidence suggests that inflammatory signaling pathways may also contribute to the pathology of DBA [17] as Iskander et al. found elevated levels of IFN-γ and TNF-α in bone marrow plasma, known instigators of stress erythropoiesis (Figure 2d). 

In conclusion, the multifaceted nature DBA presents a complex puzzle for researchers. The intricate interplay between mutations in RP genes, disruptions in ribosome assembly, and subsequent impairment of erythropoiesis underscores the need for continued investigation.

## 4. Existing Treatment Options for DBA

**Figure 3:**
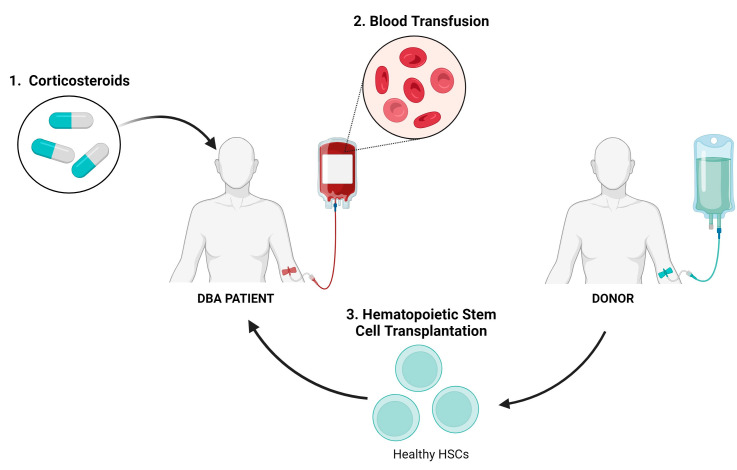
Existing treatment options for DBA patients. Created with BioRender.com (accessed on 18 April 2024).

The primary therapeutic options for anemia in DBA are the use of glucocorticosteroids, red blood cell transfusions, and hematopoietic stem cell transplantation (HSCT) (Figure 3). Vlachos and Muir et al. provide a comprehensive guide on how to treat DBA [7]. However, in this review we will emphasize mostly the limitations of these treatments and why it is necessary to develop gene therapy.

Glucocorticosteroids (GCs) serve as the primary treatment for DBA, yet their precise mechanism of action in DBA remains unclear [1]. They seem to exert a non-specific anti-apoptotic effect on erythroid progenitor cells [5,18]. Initially, approximately 80% of patients show a positive response to steroid therapy. However, approximately half of these individuals discontinue treatment due to either a loss of response or severe side effects [1,19]. These adverse effects may include growth retardation, increased risk of heart disease, osteoporosis, and severe infections [1]. Only approximately 20% of patients are able to fully discontinue steroid treatment without experiencing a relapse of anemia, achieving a state referred to as “remission” [6]. Given the profound impact of GCs on growth, physical, and neurocognitive development, the initiation of steroid administration in infants is carefully delayed, if feasible, and is maintained with chronic transfusion therapy until the child reaches one year of age [7]. For patients who do not respond to corticosteroids, blood transfusions are administered as an alternative treatment. However, a significant drawback of this approach is the potential toxicity associated with iron overload. Consequently, patients require intensive chelation therapy to mitigate the risks posed by excessive iron accumulation [1,5].

HSCT stands as the sole curative option for DBA typically recommended when resistance to corticosteroid therapy and dependence on transfusions occur [1,7]. HLA-matched sibling HSCT has demonstrated significant success rates, particularly in patients younger than 9 years old. However, each potential sibling donor undergoes thorough screening for DBA mutations, even in the absence of hematological or physical DBA manifestations [7]. Despite the efficacy of this approach, the availability of HLA-matched donors is not always guaranteed. HSCT carries several drawbacks, including the risk of graft-versus-host disease (GvHD), adverse effects stemming from preconditioning, the possibility of undetected mutations in silent carriers, and the necessity for immunosuppressive therapy post-transplantation [1,5,19].

## 5. Gene Therapy for DBA—From Research Now to Clinic in the Future

Utilizing autologous HSCT with genetically modified hematopoietic stem and progenitor cells (HSPCs) presents a potential solution to address the limitations associated with allogeneic HSCT. This innovative approach could circumvent challenges such as the scarcity of suitable donors, the risk of GvHD, the potential for graft rejection, and the possibility of donors being silent carriers of DBA mutations.

### 5.1. Lentiviral Vectors as a Potential Gene Therapy Approach for DBA

RP-mutations are the primary cause of DBA. Consequently, gene therapy aimed at enabling the expression of the functional RP gene represents a potential solution for DBA patients. Lentiviral vectors (LVs) have emerged as effective delivery tools for hematopoietic stem and progenitor cells (HSPCs) [20]. When pseudotyped with the vesicular stomatitis virus G protein (VSV-G), LVs demonstrate their versatility by transducing a wide array of cells [21]. Their large genetic capacity (up to 10 kb) and the ability to transduce both dividing and non-dividing cells make them exceptional tools for gene therapy [22]. Traditional LVs integrate the viral genome into the host’s genome, ensuring stable expression of the gene of interest [22]. The general strategy involves developing lentiviral vectors that encode the various functional RP genes mutated in DBA patients. The effectiveness of any LV-based gene therapy hinges on the successful high-level transduction of patient HSPCs that are capable of long-term hematopoietic repopulation [23]. Upon integration into the patients’ HSPCs, these cells would begin producing functional ribosomal proteins, facilitating normal erythropoiesis. However, LVs also carry oncogenic potential, as integration can occur at multiple sites, potentially leading to the disruption of normal gene function, activation of oncogenes or inactivation of tumor suppressor genes [24]. Notably, there have been several adverse events observed in clinical trials attributed to insertional mutagenesis, wherein the integration of the vector may disrupt normal genomic function or even activate oncogenes, potentially leading to adverse outcomes [24,25].

The RPS19 gene stands out as the most frequently mutated gene among individuals diagnosed with DBA, affecting approximately 25% of patients [26]. Consequently, research in this field has been directed towards elucidating methods to restore the protein encoded by this gene, aiming to reverse the hematological abnormalities associated with DBA. In a pivotal study conducted by Hamaguchi et al., the potential of gene transfer techniques to address RPS19-related pathology was demonstrated [27]. Specifically, the researchers utilized lentiviral vectors to introduce the RPS19 gene into hematopoietic progenitors from RPS19-deficient DBA patients. Remarkably, this intervention resulted in notable improvements in CD34^+^ cell proliferation, as well as in erythroid development. Further supporting the feasibility of gene transfer as a therapeutic strategy for DBA, additional studies have corroborated these findings. For instance, Jaako et al. used transgenic mice containing a RPS19-targeting shRNA under a doxycycline-responsive promoter for lentiviral-based gene therapy. They transduced uninduced BM cells from heterozygous (D/+) and homozygous (D/D) RPS19-targeting shRNA DBA mice with lentivirus containing RPS19 cDNA and transduced cells were transplanted into wild type mice. Following engraftment, the mice were administered doxycycline to downregulate the endogenous RPS19 and induce the disease. They concluded that enforced expression of RPS19 cures anemia and prevents fatal bone marrow failure in RPS19-deficient mice. Additionally, they observed that cells corrected with the RPS19 gene displayed sustained improvement in pan-hematopoietic function over time, contrasting with untreated cells, and showed no adverse effects attributable to the gene transfer process [28]. Following this study, Debnath et al. engineered lentiviral vectors capable of expressing the RPS19 gene under the control of the human elongation factor 1α short (EFS) promoter, a clinically relevant promoter [29]. To evaluate the efficacy of this vector, they transfected c-Kit-enriched BM cells from both control and heterozygous RPS19 shRNA mice in the presence of doxycycline, and subsequently injected these cells into lethally irradiated wild type mice. Their results revealed that recipients transduced with EFS-RPS19 shRNA BM exhibited near normal blood cellularity, indicating that enforced expression of RPS19 driven by the EFS promoter can effectively treat severe anemia and bone marrow failure in RPS19-deficient mice. However, it is worth noting that this model does not mimic the haploinsufficiency seen in DBA patients, which is caused by mutations in the RPS19 gene. More recently, this group designed a clinically applicable self-inactivating (SIN) lentiviral vector containing the human RPS19 driven by the human EFS promoter for the clinical development of gene therapy for RPS19-deficient DBA patients. Their study showcased that this vector effectively rescues the anemia and lethal BM failure phenotype observed in the mouse models of RPS19-deficient DBA, with low risk of mutagenesis and a highly polyclonal insertion site pattern. Additionally, they observed the restauration of impaired erythroid differentiation in human RPS19-deficient CD34+ cord blood cells treated with this vector, underscoring its potential for clinical translation and therapeutic benefit in DBA patients [30].

Furthermore, the exploration of using lentiviral vectors to express GATA1 for the promotion of red blood cell production is under investigation. This approach offers significant advantages, particularly in targeting the majority of DBA mutations rather than a specific one. In vitro studies have shown that overexpression of GATA1 in hematopoietic stem and progenitor cells (HSPCs) from DBA patients can rescue erythroid differentiation defects [1,31]. While gene therapy presents an attractive strategy for curing DBA, the traditional approach of overexpressing a functional copy of a mutated gene is not the most efficient. This is because it would necessitate the development and validation of numerous gene therapy vectors, each containing a copy of one of the mutated DBA genes. Instead, a unified gene therapy strategy is being proposed, which involves the developmentally regulated and highly restricted expression of GATA1. This strategy is anticipated to be curative for most, if not all, DBA patients, regardless of the specific mutation causing the disease [31].

While extensive research has been conducted on the utilization of LVs for gene therapy in DBA, these efforts have not yet been translated into clinical trials. Nevertheless, the successful implementation of LVs in gene therapy for various genetic blood disorders, including sickle cell disease (SCD), β-thalassemia, and Fanconi anemia (FA), underscores the potential of LV-based gene therapy as a promising avenue for treating DBA effectively (Table 1).

### 5.2. CRISPR/Cas9 Non-Integrating Lentiviral-Based Gene Therapy

Another strategy relies on non-integrating lentiviruses (NILVs) offering two primary methodologies for generating non-integrating lentiviral vectors: (i) introducing mutations in the viral integrase protein, and (ii) inhibiting the recognition of viral DNA by this enzyme through mutations in the sites [49,50]. Gurumoorthy et al. summarize the point mutations that have been used to develop NILVs [50]. By employing NILVs, the viral genome can persist in the host cell as an episome, rather than integrating into the host genomic DNA [51]. This mechanism creates an opportunity for the application of CRISPR/Cas9 technologies. Once the desired DNA editing event occurs, the Cas9 protein and the guide RNA (gRNA) are no longer required for ongoing transgene expression. Therefore, they can be removed from the cell, minimizing the risk of off-target effects and undesirable side effects associated with their continued presence [49]. The difference between the traditional integrating LVs and NILVs is highlighted in Table 2.

CRISPR/Cas9 technology has revolutionized the field of gene editing, offering a highly precise and efficient method for modifying the genome. This breakthrough has paved the way for innovative approaches in addressing genetic disorders. Alongside these benefits come significant ethical and safety concerns. One of the primary concerns is the risk of off-target effects, wherein the CRISPR/Cas9 mechanism may unintentionally alter DNA sequences at unintended sites, resulting in unintended mutations and the activation of oncogenes [53]. Oncogenic changes created by these mechanisms will be carried by the target cell and its progeny, adding another layer of complexity to the potential long-term impacts of gene therapy technologies [24]. Additionally, the induction of double-strand breaks (DSBs) leads to genomic instability and can consequently lead to more accumulation of mutations. DSBs are primarily repaired by the error-prone non-homologous end joining (NHEJ) pathway, which can lead to small insertions and deletions (INDEL mutations) [54]. Such occurrences could potentially give rise to unforeseen health issues or even the emergence of novel diseases.

One of the key advancements in the field of gene therapy is the development of base and prime editors, which are tools that can be used to correct mutations in DNA. While CRISPR/Cas9 genome editing conventionally triggers DSBs at specific DNA target sites, potentially resulting in genomic instability and off-target effects, base editors (BE) and prime editors (PE) use Cas9 nickases (dCas9), which are variants of the Cas9 that have been engineered to induce nicks in the DNA strand instead of cleaving it [55]. Table 3 lists the main differences between the traditional CRISPR/Cas9 system, base editors, and prime editors.

In the context of base editors, dCas9 is combined with a deaminase enzyme, enabling the alteration of a single DNA through a process called deamination [59]. Given that approximately half of all known pathogenic genetic variants are single nucleotide variants (SNVs), base editing represents a promising approach for treating a wide range of genetic diseases [60]. There are two classes of DNA base-editors: cytosine base-editors (CBE) and adenine base-editors (ABE) [55]. CBEs convert cytosine bases to uracil which is then recognized by the cell’s replication machinery as thymine, leading to a C-G to T-A substitution in the DNA sequence. This mechanism is particularly useful for correcting disease-causing mutations that involve C-G to T-A changes. However, the efficiency of CBE in human cells has been limited due to the cellular repair pathway that reverts the uracil to cytosine, known as base excision repair (BER). To overcome this, researchers have developed improved versions of CBE, such as BE2 and BE3, which incorporate strategies to inhibit BER, thereby enhancing the editing efficiency and specificity of CBE [55]. ABEs, on the other hand, convert adenine to inosine, which is then recognized as guanine during DNA replication, leading to the substitution of A-T to G-C in the DNA [55], which represent the most common pathogenic SNVs reported in ClinVar database [60]. However, base editors are not without limitations. Besides being restricted to making a maximum of four substitutions, the limitations include the requirement for precise positioning of the base editor edit window, and off-target DNA and RNA editing [57].

The prime editor system is a sophisticated genetic editing tool that allows for precise modifications of DNA, including point mutations, insertions, and deletions of small fragments [58]. This system is composed of two main components: a reverse transcriptase (RT) and a Cas9 nickase fused together. The RT component is guided by a prime editing guide RNA (pegRNA), which contains a primer binding site (PBS) and a template for the reverse transcription. This process is facilitated by dCas9, which exposes the 3′ end of the DNA strand, allowing the RT to bind to the PBS on the pegRNA and synthesize the new DNA strand with the desired edit. The edited DNA strand then has two overhangs: one unmodified 5′ flap and one purposed 3′ flap. The 3′ flap, which contains the desired edit, is retained, while the 5′ flap is cleaved away. The cellular DNA repair system then integrates the edited DNA strand into the genome, replacing the original sequence with the modified version [55,58]. To enhance efficiency, an improved version, PE3, was developed. This version incorporates a second nicking guide RNA (ngRNA) to induce a nick in the non-edited strand. This process leads to the corrected strand being used as a template for correction of the nicked, resulting in incorporation of the desired change in both strands. However, this approach also increases the number of insertions and deletions (INDELs). To mitigate this issue, the PE3b version was introduced. This version utilizes a ngRNA that specifically recognizes the non-edited strand after the edit has occurred, thereby enhancing the safety of the editing process [56].

Although the PE system offers enhanced flexibility in targeting compared to other genome editing methods, such as BE, it faces several practical limitations. These include genomic scope constraints, variable efficiency across cell types, and delivery challenges due to its original large size [58]. To address the genomic scope constraints, researchers have focused on developing PAM-relaxed Cas9 enzymes that are compatible with various protospacer adjacent motif (PAM) sequences, beyond the traditional SpCas9 which requires the NGG PAM [58]. The NGG motif represents a particular three-nucleotide sequence (NGG), where N can represent any base. This sequence is what the SpCas9 enzyme identifies and attaches to, enabling it to sever the DNA at the desired location [61,62].

Additionally, researchers have focused on developing miniaturized Cas9 versions to potentially reduce the size of the PE system and facilitate its delivery [58]. The size limitation is of particular importance when developing NILVs that carry the prime editor system. As mentioned before, a typical lentiviral vector can carry up to 10 kb of insert fragments [22]. For prime editing, the lentiviral vector must include the PE components, including the dCas9 (~4.2 kb) and the RT (~2.0 kb), with a total size of approximately 6.2 kb [58]. While NILVs can offer advantages in gene therapy applications, the capacity of the lentiviral vector may exceed when including both PE components and the pegRNA, along with promoters, which further states the need to use miniaturized Cas9 versions. An alternative approach involves the delivery of the Cas9 protein along with NILVs that carry the gRNA. This method has been successfully demonstrated by Uchida et al., who developed a Cas9 delivery system using NILVs that encode both a gRNA and a donor template for correction of the sickle cell disease mutation [63]. This system erases the possibility of exceeding the lentiviral vector size capacity and could be adapted for prime editing.

While non-integrating lentivirus still requires further development to reach clinical trials, the successful application of gene therapy using the CRISPR/Cas9 tool in treating genetic blood disorders like SCD and β-thalassemia has sparked optimism for correcting mutations associated with DBA through gene therapy (Table 4).

This breakthrough paves the way for exploring similar approaches to target and rectify DBA mutations, offering hope for more effective treatment options and potentially even a cure for this rare hematological disorder [77,78]. Additionally, recent research highlights the effective use of prime editing to correct the HBB gene in hematopoietic stem cells (HSCs) of mice with sickle cell disease (SCD). This innovative approach utilized a HDAd vector to deliver the prime editing machinery directly into the bloodstream of the mice, showcasing the potential for in vivo gene correction as a promising therapeutic strategy for genetic blood disorders like SCD [79].

Furthermore, beyond the numerous ongoing and completed clinical trials, FDA has approved two gene therapies for the treatment of SCD: Casgevy and Lyfgenia. Casgevy employs a novel genome editing technique that modifies a particular gene to restore the production of fetal hemoglobin, thereby mitigating the abnormal red blood cells typical of SCD. On the other hand, Lyfgenia employs a lentiviral vector to deliver a healthy hemoglobin-producing gene to patients, aiming to correct the underlying genetic defect causing SCD [80,81]. Additionally, there is also an FDA-approved gene therapy for β-Thalassemia. Zynteglo utilizes a replication-incompetent lentiviral vector to deliver a modified β-globin gene to the patient’s own hematopoietic stem cells (HSCs). This approach allows to produce functional adult hemoglobin, addressing the underlying genetic cause of β-thalassemia by correcting the α/β-globin imbalance [81,82].

Despite the promising advancements in gene therapy, significant challenges persist, particularly in the areas of long-term patient follow-up, cost, safety, efficacy, and manufacturing. Ensuring the long-term safety of gene therapy products necessitates extensive follow-up beyond the active clinical trial period to monitor for delayed adverse effects [83]. Additionally, HSCT continues to present significant challenges, including its high cost, inherent safety concerns, variability in efficacy, and manufacturing difficulties. A key challenge lies in the production of therapeutic agents at high titers and with consistent quality [84].

**Figure 4:**
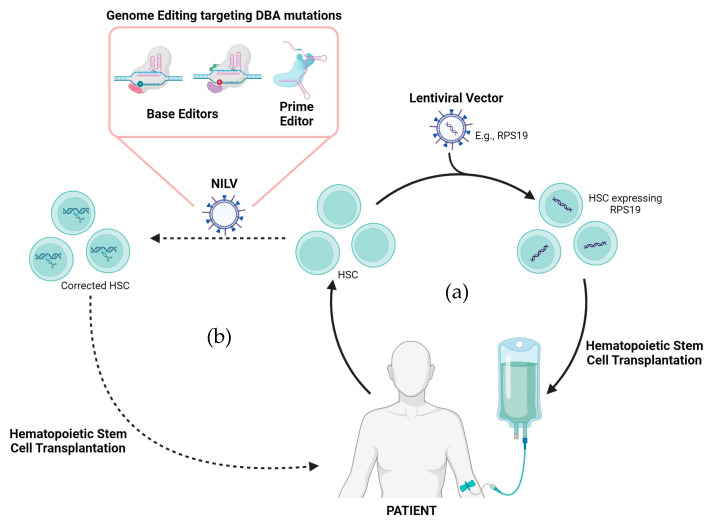
Gene therapy as a therapeutic alternative for DBA treatment. (**a**) Traditional gene therapy employes integrating lentiviral vectors carrying the functional gene (e.g., RPS19), which are delivered to the patients HSCs ex vivo. (**b**) A more novel approach includes correcting the mutation ex vivo using non-integrating lentiviral vectors (NILVs) that carry CRISPR/Cas9 tools specific for the DBA mutation. Created with BioRender.com (accessed on 18 April 2024).

Despite these challenges, advancements in gene therapy, particularly using lentivirus and CRISPR/Cas9 tools, have demonstrated significant potential in treating various blood diseases. These innovative technologies offer a promising future for DBA treatment by providing precise and targeted corrections to genetic defects (Figure 4). These advancements not only offer a safer and more effective alternative to traditional treatments like allogenic HSCT but also hold the promise of a long-term cure for DBA and other monogenic diseases. Further research and clinical trials are necessary to fully realize the potential of these gene therapy approaches and to address the unique challenges posed by DBA, such as the involvement of multiple genes and the unknown causative mutation in some patients.

## 6. Conclusions

The utilization of autologous HSCT combined with genetically modified HSPCs presents a promising alternative to overcome the limitations associated with allogenic HSCT and represents a promising leap forward in addressing the challenges associated with DBA treatment. Through targeted research efforts focused on restoring the function of the RPS19 gene, frequently mutated in DBA patients, and the development of innovative gene therapy techniques such as base and prime editing using NILVs, significant progress can be made towards effective therapeutic interventions. However, it is important to note that autologous HSCT is associated with high costs, which can be a limiting factor for its widespread adoption. Despite these challenges, the potential benefits of this approach including improved outcomes, reduced risks, and the potential for long-term remission, make it a compelling option for treating genetic blood diseases like DBA.

## Figures and Tables

**Table 1:** An overview of clinical trials utilizing LVs for gene therapy of genetic blood disorders.

Disease	Clinical Trial ID	Intervention/Treatment	**Ref.**
**Sickle Cell Disease (SCD)**	NCT02186418	CD34+ cells transduced with gamma-globin lentiviral vector.	[32]
	NCT03282656	Single infusion of autologous bone marrow derived CD34+ HSC cells transduced with the lentiviral vector containing a short-hairpin RNA targeting BCL11a.	[33]
	NCT05353647	Autologous transplantation of CD34+ HSC cells transduced with the lentiviral vector containing a shRNA targeting BCL11a.	[34]
	NCT02247843	Autologous transplantation of peripheral blood CD34+ cells transduced ex vivo by the Lenti/G-βAS3-FB lentiviral vector to express an anti-sickling (βAS3) gene.	[35]
	NCT03964792	Transplantation of an autologous CD34+ enriched cell fraction that contains CD34+ cells transduced ex vivo with the GLOBE1 lentiviral vector expressing the βAS3 globin gene (GLOBE1 βAS3 modified autologous CD34+ cells).	[36]
**SCD and β-Thalassemia**	NCT02151526	Administration of LentiGlobin BB305 drug product to participants with either transfusion dependent β-thalassemia (TDT) or sickle cell disease (SCD).	[37]
**β-Thalassemia**	NCT03276455	Autologous transplantation of HSCs transduced with lentiviral vector encoding for beta-globin gene.	[38]
	NCT02453477	Autologous transplantation of HSCs genetically modified with GLOBE lentiviral vector encoding for the human beta-globin gene.	[39]
	NCT06219239	Autologous transplantation of HSCs transduced with lentiviral vector encoding βA-T87Q-globin gene.	[40]
	NCT05745532	Autologous transplantation of HSCs transduced with LentiHBBT87Q system to restore β-globin expression.	[41]
	NCT06280378	Autologous transplantation of CD34+ stem cells transduced ex vivo with a lentiviral vector encoding βA-T87Q-globin.	[42]
	NCT01639690	Autologous transplantation of CD34+ cells transduced with TNS9.3.55 lentiviral vector encoding the normal human ß-globin gene.	[43]
	NCT05762510	Autologous transplantation of CD34+ HSCs transduced with LentiRed lentiviral vector.	[44]
	NCT05757245	Autologous HSCT using GMCN-508A drug product (autologous CD34+ HSCs transduced with GMCN-508A lentiviral vector encoding the human α-globin gene).	[45]
	NCT05015920	Transplantation of autologous CD34+ stem cells transduced with a lentiviral vector encoding βA-T87Q-globin.	[46]
**Fanconi** **Anemia (FA)**	NCT01331018	Transplantation of autologous patient blood stem cells that have been corrected in the laboratory by introduction of the normal FANCA gene.	[47]
	NCT04437771	Transplantation of autologous CD34+ cells transduced with lentiviral vector carrying the FANCA gene.	[48]

**Table 2:** Main characteristics, applications, and limitations of integrating LVs and NILVs.

Aspect	Integrating Lentiviral Vectors (ILVs)	Non-Integrating Lentiviral Vectors (NILVs)
**Integration**	Integrates the transgene into the host genome [22,50]	Does not integrate the transgene into the host genome [52] Expresses the transgene from episomal DNA in non-dividing cells or transiently in dividing cells [49]
**Expression**	Stable integration of the transgene into the host genome [49,50]	Enables transient expression or sustained episomal expression [50]
**Safety**	Higher risk of insertional mutagenesis and malignant transformation [50]	Reduced risk of insertional mutagenesis and malignant transformation [50]
**Applications**	Gene therapy for long-term gene expression [50,52] Recombinant protein production [50] Vaccination [50] Cell imaging [50]	Gene therapy for mutation correction [50,52] Cytotoxic cancer therapies [49,50] Cellular differentiation [49] Vaccination [49,52] Immunotherapies [49,50]
**Limitations**	Insertional mutagenesis [24] Oncogenic potential [24]	Residual integration risks [50] Transient expression is not suitable for all applications

**Table 3:** Comparison between CRISPR/Cas9 system, base editors, and prime editor. CBE: cytosine base editor; ABE: adenine base editor; PE: prime editor.

Tools	Components	Applicability	Advantages	Drawbacks	Ref.
**CRISPR/Cas9**	Cas9, sgRNA, and donor DNA (for HDR)	Point mutations Large DNA insertions and deletions Gene knock-out	Versatility in gene insertion, deletion, and modification	DSB induction Off-target effects Low efficiency for HDR	[56]
**CBE**	dCa9-cytosine deaminase, and sgRNA	Transitions mutations (C→T, G→A, A→G, T→C)	Induction of SSBs	Requires precise positioning of editing window Off-target DNA and RNA editing Bystander edits Only capable of four transition mutations	[56,57]
**ABE**	dCas9-adenine deaminase and sgRNA				
**PE**	dCas9(H840A)-M-MLV RT and pegRNA	Point mutations Small deletions and insertions	Induction of SSBs Allows for all precise modifications	Genomic scope constraints Variable efficiency in different cell types Delivery challenges due to large size of PE	[56,58]

**Table 4:** An overview of clinical trials utilizing CRISPR/Cas9 technology for gene therapy of genetic blood disorders.

Disease	Clinical Trial ID	Intervention/Treatment	Ref
**Sickle Cell Disease (SCD)**	NCT06287099	Autologous CRISPR/Cas9 modified CD34+ hHSPCs (BRL-101)	[64]
	NCT04819841	Gene correction in autologous CD34+ HSCs (HbS to HbA) to treat severe SCD	[65]
	NCT03745287	Autologous CRISPR/Cas9 modified CD34+ hHSPCs using CTX001	[66]
	NCT05951205	Single dose of CTX001 in subjects with severe SCD with βS/βC genotype	[67]
	NCT04774536	Transplantation of CRISPR/Cas9 corrected HSCs (CRISPR_SCD001) in patients with severe SCD	[68]
	NCT05329649	Administration of a single dose of CTX001 in pediatric subjects with severe SCD	[69]
**SCD and β-Thalassemia**	NCT05477563	Single dose of autologous CRISPR/Cas9 modified CD34+ hHSPCs (CTX001) in subjects with transfusion-dependent β-Thalassemia or severe SCD	[70]
	NCT04208529	A long-term follow-up study of subjects with β-thalassemia or SCD treated with autologous CRISPR/Cas9 modified HSCs (CTX001)	[71]
**β-Thalassemia**	NCT03655678	Autologous CRISPR/Cas9 modified CD34+ hHSPCs using CTX001 in subjects with transfusion-dependent β-Thalassemia	[72]
	NCT04925206	Autologous CRISPR/Cas9 modified CD34+ hHSPCs using ET-01 in subjects with transfusion-dependent β-Thalassemia	[73]
	NCT05577312	Autologous CRISPR/Cas9 modified CD34+ hHSPCs (BRL-101)	[74]
	NCT05356195	Autologous CRISPR/Cas9 modified CD34+ hHSPCs (CTX001) in pediatric subjects with transfusion-dependent β-Thalassemia	[75]
	NCT03728322	Gene correction of HBB in patient-specific iHSCs using CRISPR/Cas9	[76]
